# The Role of Vitamin D in Alzheimer’s Disease: A Transcriptional Regulator of Amyloidopathy and Gliopathy

**DOI:** 10.3390/biomedicines10081824

**Published:** 2022-07-28

**Authors:** Jiseung Kang, Mincheol Park, Eunkyung Lee, Jieun Jung, Tae Kim

**Affiliations:** Department of Biomedical Science and Engineering, Gwangju Institute of Science and Technology, Gwangju 61005, Korea; wltmd1006@gist.ac.kr (J.K.); eyejor@gist.ac.kr (M.P.); eunkyung9732@gm.gist.ac.kr (E.L.); jje90626@gist.ac.kr (J.J.)

**Keywords:** Alzheimer’s disease, vitamin D, amyloidopathy, gliopathy, mouse, memory impairment

## Abstract

Alzheimer’s disease (AD) is characterized by amyloid-beta (Aβ) accumulation and cognitive mental decline. Epidemiological studies have suggested an association between low serum vitamin D levels and an increased risk of AD. Vitamin D regulates gene expression via the vitamin D receptor, a nuclear ligand-dependent transcription factor. However, the molecular mechanism underlying the pathogenic and therapeutic effects of vitamin D on AD is not fully understood yet. To better understand how vitamin D regulates the expression of genes related to AD pathology, first, we induced vitamin D deficiency in 5xFAD mice by providing a vitamin-D-deficient diet and observed the changes in the mRNA level of genes related to Aβ processing, which resulted in an increase in the Aβ load in the brain. The vitamin D-deficient diet also suppressed the expression of genes for microglial Aβ phagocytosis. Interestingly, vitamin D deficiency in the early stage of AD resulted in earlier memory impairment. In addition, we administered vitamin D intraperitoneally to 5xFAD mice with a normal diet and found lower Aβ levels with the suppressed expression of genes for Aβ generation and observed improved memory function, which may be potentially associated with reduced MAO-B expression. These findings strongly suggest the role of vitamin D as a crucial disease-modifying factor that may modulate the amyloid pathology with regard to reducing AD symptoms.

## 1. Introduction

Alzheimer’s disease (AD) is a chronic neurodegenerative disorder pathologically defined by the extracellular accumulation of amyloid-beta (Aβ) and intracellular neurofibrillary tangles and is characterized by deteriorating memory and cognition [1]. Amyloid-beta (Aβ) has been implicated in initiating pathophysiological changes that lead to cognitive impairment and neurodegeneration by the interruption of axonal transport, neurotrophic factor synthesis, and neuronal signal pathways [2].

Regulating the Aβ pathway may be a beneficial therapeutic target for AD treatment or prevention, and this pathway may be attenuated by the vitamin D levels, as there is numerous epidemiologic evidence to support the pathological role of vitamin D deficiency in the development of AD, including prospective cohort studies and meta-analyses [3,4,5,6,7,8,9]. Numerous studies have shown that neurodegenerative diseases like multiple sclerosis, Parkinson’s disease, and AD are related to vitamin D deficiency [10,11,12].

Vitamin D is a fat-soluble steroid hormone necessary for bone and muscle health. However, the discovery of vitamin D’s autocrine pathways in various cell types has prompted an interest in its role in central nervous system (CNS) functions [13,14]. Vitamin D receptors (VDR) are found in the cerebral cortex and hippocampus, which are critical to cognition and memory [15]. In addition, vitamin D has been linked to neuroprotection and cognitive function in aging brains [16], and its deficiency has been related to memory impairment in the Morris water maze test in aging rats [17]. Although the correlations between AD and low serum vitamin D levels have been studied extensively, the molecular mechanisms are not yet fully understood.

Vitamin D needs to be converted to bioactive 1,25-dihydroxyvitamin D, and this conversion is catalyzed by 25-hydroxylase and 1α-hydroxylase. VDR and 1α-hydroxylase are distributed in the neurons and glia of the human brain [18], and vitamin D and its metabolites can cross the blood–brain barrier [19]. Thus, vitamin D can be metabolized locally in the central nervous system (CNS). In the CNS, vitamin D performs genomic actions as a transcriptional factor and is mediated by the interaction with the VDR/RXR (retinoic acid receptor) complex [20]. The vitamin D-binding VDR/RXR complex in the cell nucleus regulates various gene expressions by binding the vitamin D responsive elements (VDREs) located in the regulatory regions to promote gene expression [20]. Therefore, vitamin D deficiency is implicated in modulating CNS functions by regulating gene expression.

As one of the major causes of AD, amyloidopathy is regulated by several processes, including generation, degradation, and microglial and astrocytic clearance of Aβ. Various enzymes are involved in the pathways for Aβ plaque formation and degradation. Aβ plaques contain Aβ peptides synthesized by the proteolytic amyloidogenic processing of the amyloid precursor protein (APP). Aβ generation depends on the sequential cleavage of APP by β-secretase (BACE) and γ-secretase [21]. γ-secretase is a heterotetrameric protein complex [22] consisting of presenilin 1 or 2 (PS1 or PS2) [23], nicastrin (Ni), anterior-pharynx-defective 1a or 1b (Aph1a or Aph1b), and presenilin-enhancer 2 (PEN2) [24]. Along with the amyloidogenic pathways, some members of the A Disintegrin and Metalloproteinase (ADAM) family, such as ADAM10, have been shown to have α-secretase activity [25]. Thus, α-secretase competes with amyloidogenic Aβ formation. The Aβ levels are determined not only by the proteolytic activities of APP-cleaving secretases but also by the degradation of Aβ via endopeptidases, such as neprilysin (NEP) and the insulin-degrading enzyme (IDE) [26]. In addition, 1,25-dihydroxyvitamin D regulates the expression of the genes involved in the Aβ processing pathway in neuronal cultures [24]. Thus, vitamin D deficiency may disrupt normal gene expression and lead to AD pathology.

Besides Aβ pathology, AD is also associated with chronic neuroinflammation, as well as gliopathy, involving microglial and astrocytic changes. Vitamin D_3_ affects microglia immune activation by increased interleukin-10 (IL-10) expression [27], and VDR activation regulates microglia polarization and oxidative stress [28]. A bioactive form of vitamin D_3_ (1,25-dihydroxyvitamin D_3_) prevents neuroinflammation [29]. However, the prolonged activation of astrocytes creates a neurotoxic environment, which could exacerbate the progression of AD [30]. Reactive astrocytes induce an augmenting inflammatory response in neurodegenerative disorders [31], and vitamin D_3_ administration represses astrocyte activation [32]. Although past research indicates that vitamin D may modulate gene expression related to glial functions, there is insufficient evidence. Therefore, we sought to understand how vitamin D deficiency alters gene expression and affects glial function by using an AD mouse model to mimic human pathology. In addition, we sought to elucidate the potential therapeutic mechanisms of vitamin D as a crucial disease-modifying factor that may regulate amyloid pathology.

## 2. Materials and Methods

### 2.1. Animals

Experiments were carried out using male transgenic hemizygous 5xFAD mice (Jackson Laboratory, Bar Harbor, ME, USA) that overexpressed both the human APP gene with Swedish, Florida, and London mutations and the human PS1 gene with mutations M146L and L286V [33]. All animal protocols were approved by the ethics committee at Gwangju Institute of Science and Technology, following the Institutional Animal Care and Use Committee guidelines.

The 5xFAD mice were randomly assigned to eight groups (*n* = 6/group) that included: (1) The Early-Control group received a normal diet (RodFeed, DBL, Eumseong, Korea) that was manufactured based on NIH-41 Open Formula (20 % crude protein, 4 % crude fat, 62 % carbohydrate, and 8 IU/kg vitamin D3), consistently. (2) The Early-Deficiency group was fed a vitamin D-depleted diet (TD. 89123, ENVIGO, Indianapolis, IA, USA) from the age of 1 month. A vitamin D-depleted diet was similarly formulated, except without vitamin D3 (16 % crude protein, 10 % fat, 59 % carbohydrate, and 0 IU/kg vitamin D3). (3) The Late-Control group consistently received the normal diet. (4) The Late-Deficiency group was fed a vitamin D-depleted diet from the age of 3 months. (5) The Intermediate-Vitamin D supplementation group was fed the normal diet and intraperitoneally injected each week with 410 ng/g cholecalciferol (PHR1237, Sigma-Aldrich, Burlington, MA, USA) in saline containing 1% ethanol starting at 3 months old. (6) The Intermediate-Vehicle group was fed the normal diet and injected intraperitoneally only saline containing 1 % ethanol (injection volume: individual body weight (g)/100 mL saline) starting at 3 months old. (7) The Late-Vitamin D supplementation group was fed the normal diet and intraperitoneally injected each week with 410 ng/g cholecalciferol (PHR1237, Sigma-Aldrich) in saline containing 1 % ethanol starting at 4.5 months old. (8) The Late-Vehicle group was fed the normal diet and injected intraperitoneally with only saline with 1 % ethanol (injection volume: individual body weight (g)/100 mL saline) starting at 4.5 months old.

We determined the vitamin D injection dose for mice within a safe range against hypervitaminosis D. Given that the human vitamin D injection dose is 300,000 IU/month [34], we calculated the safe dosage of vitamin D with the formula for dose conversion between animal and human studies [35]. All the mice were supplied with food and water ad libitum, with a constant temperature and humidity. Before the behavioral tests and molecular analysis, mice undergwent an induction period of at least six weeks to induce vitamin D deficiency. There was no difference in daily food intake between the Early-Con and Early-Def groups, but the Early-Def group had more weight gain (Figure S1).

### 2.2. Tissue collection

Animals were deeply anesthetized using isoflurane and transcardially perfused with 1X PBS. Brains were harvested and dissected into two hemispheres. The right hemisphere was dissected to separate the prelimbic area, frontal cortex, thalamus, hippocampus, pons, and cerebellum and then stored at −80 °C until use.

### 2.3. ELISA

The cortical, hippocampal region, and pons were individually homogenized with a disposable polypropylene pellet pestle in 800 μL of 20 mM Tris-HCl and 5 mM EDTA (pH 7.8), with a protease inhibitor cocktail (P3100, GenDEPOT, Katy, TX, USA). The total protein of the homogenized tissue was quantified with a Micro BCA^TM^ Protein Assay kit (23225, Thermo Fisher Scientific, Waltham, MA, USA). The homogenates were centrifuged at 430,000 rcf for 20 min in a Beckman TLA 100.3 centrifuge rotor (Beckman Coulter, Fullerton, CA, USA) at 4 ℃, and the soluble Aβ supernatant was collected. The remaining insoluble pellets were rehomogenized in 800 μL of 5 mM GHCl and 50 mM Tris-HCl (pH 8.0) with a pellet pestle. This solution was shaken for 4 h and then centrifuged at 430,000 rcf for 20 min in a Beckman TLA 100.3 centrifuge rotor. Afterward, the supernatants were collected. ELISA was conducted for soluble and insoluble Aβ 42 following the manufacturer’s instructions (KHB3441, Thermo Fisher Scientific, Waltham, MA, USA).

### 2.4. qRT-PCR

Following the manufacturer’s instructions, TRI reagent (TR118, Molecular Research Center, Cincinnati, OH, USA) was used to extract the total RNA from all right hemisphere tissues, including, but not limited to, the prelimbic area, hypothalamus, and cerebellum regions, following dissection of the brain regions for Aβ measurement. RNA (2.5 μg) was reverse-transcribed into cDNA using oligo(dT)18 primers (RT200, TOPscript^TM^ RT DryMIX (dT18 plus), Enzynomics, Daejeon, Korea). Oligomer containing a reverse transcription tube was incubated at 50 ℃ for 60 min, followed by a 95 ℃ inactivation stage for 5 min. Then, a cDNA sample (1 μL) was used as a template for qPCR in each well and amplified with the primers listed in Table 1. Then, qPCR was performed with TOPreal qPCR 2x PreMIX (RT5015, Enzynomics, Daejeon, Korea) for 40 cycles, each with 60 °C annealing for 15 s and elongation at 72 °C for 30 s. The 2^−ΔΔCt^ method was used to calculate the change in the gene expression fold to measure the differentially expressed genes in each group.

### 2.5. Behavioral Test

By using standard behavioral test protocols, these three behavioral tests were performed: the open field test (OFT), the novel place recognition (NPR) test, and the elevated plus maze (EPM) test [36,37,38]. The tests were run on 4 consecutive days within the dark cycle onset (between 19 and 24 pm) in a sound-attenuated room separate from the home cage.

On the first day of testing, OFT was performed to evaluate the locomotor activity and anxiety levels of mice. The test area consisted of a 40-cm^3^ open-topped square container under red light (~50 lux). Mice were placed in the empty test area and allowed to explore freely for 10 min before returning to their home cage.

Over the next two days, a novel place recognition (NPR) test was performed to examine their hippocampus-dependent spatial memory [38]. On the second day of testing (acquisition trial), two identical objects (Object A and Object A’) were each placed in the same position relative to the visual cue, and mice were allowed to explore the two objects in the square arena with visual cues for 10 min. After 24 h, on the third day of testing (test trial), one of the objects was relocated to a novel location, and mice explored the two objects in the familiar location and novel location for 10 min. The discrimination index implies a relative preference for the novel location and is calculated by the equation below.

$$\begin{array}{l} \mathrm{Discrimination}\;\mathrm{index}\\ =\frac{Time\;spent\;in\;novel\;location-Time\;in\;familiar\;location}{Time\;spent\;in\;novel\;location+Time\;in\;familiar\;location} \end{array}$$

On the fourth and final day of testing, the EPM test was performed to measure anxiety-like behavior based on the natural aversion of mice to the open and elevated areas and their natural, spontaneous exploratory behavior in novel environments [37]. The test area consisted of four arms (25 cm long × 5 cm wide) connected to a center zone (5 × 5 cm) and elevated 50 cm above the floor under ~300 lux light. Two arms were enclosed with walls (16 cm high, closed arm), and the other arms had no border in place of the walls (open arms). Mice were placed on the open arms with their head facing toward the center zone and allowed to explore freely for 5 min. The behavioral tests were video-recorded and analyzed using automated video tracking system Smart 3 (Panlab, Harvard Apparatus, Barcelona, Spain).

### 2.6. Quantification and Statistical Analysis

All data were analyzed with GraphPad Prism version 9 (GraphPad Software, La Jolla, CA, USA) and presented as the means ± SEM (* *p* < 0.05, ** *p* < 0.01, and *** *p* < 0.001). For simple comparisons, the Student’s *t*-test was used.

## 3. Results

### 3.1. Vitamin D Deficiency Increased the Brain Aβ Load

**Figure 1:**
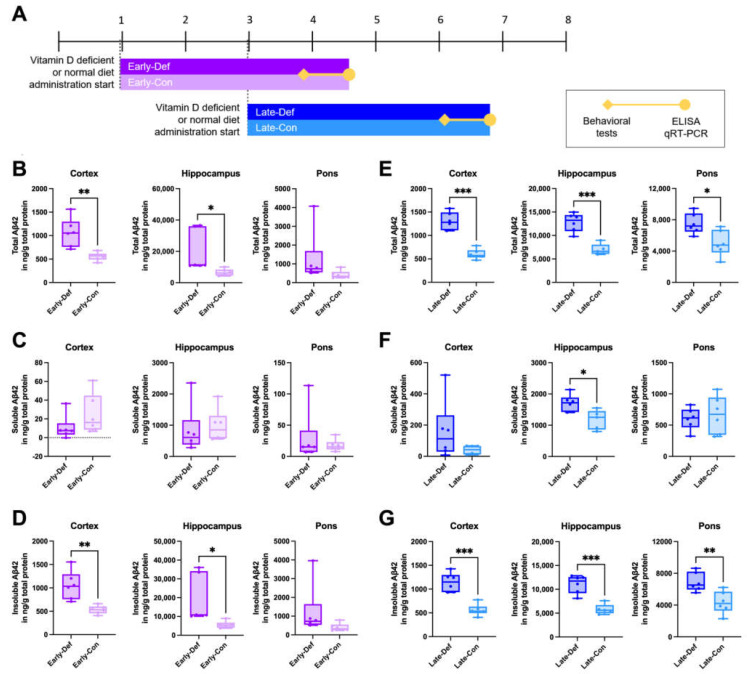
Vitamin D deficiency increased the Aβ load in both the early and late stages of AD model mice. (**A**) Experimental scheme of a vitamin D-deficient state in 5xFAD mice induced by the administration of a vitamin D-depleted (0 IU) diet. One-month-old mice in the Early-Def (*n* = 6) and Early-Con (*n* = 6) groups were given vitamin D-depleted and normal diets, respectively. Late-Def (*n* = 6) and Late-Con (*n* = 6) group mice were given a vitamin D-depleted diet and normal diet, respectively, starting from 3 months old. (**B**) The total Aβ42 levels in the cortex, hippocampus, and pons of the 4.5-month-old Early-Def and Early-Con groups were measured using ELISA kits. (**C**) Soluble and (**D**) insoluble Aβ42 levels were also represented. (**E**) The total Aβ42 levels in the cortex, hippocampus, and pons of the ~7-month-old Late-Def and Late-Con groups are indicated. (**F**) The soluble and (**G**) insoluble Aβ42 levels are also depicted. Dots represent the data of individual mice in each group. The data are presented by the mean ± SEM of each group. * *p* < 0.05, ** *p* < 0.01, and *** *p* < 0.001 by the Student’s *t*-test.

A vitamin D-deficient state was generated by administering a vitamin D-depleted (0 IU) diet in 5xFAD mice. To assess the putative stage-dependent effects of vitamin D on AD, transgenic mice were separated into two groups: before the onset of (‘early stage’) and during the developmental period of amyloid pathology (‘late stage’) (Figure 1A). We confirmed that vitamin D deficiency at an early stage induced significantly increased total Aβ42 levels in the cortex and hippocampus (Figure 1B). Total Aβ42 consists of soluble and insoluble Aβ42. There are no significant differences in the soluble Aβ42 levels between the Early-Def and Early-Con groups (Figure 1C), but Early-Def displayed significantly elevated insoluble Aβ42 levels in the cortex and hippocampal regions (Figure 1D). The Late-Def group exhibits higher total Aβ42 levels in the cortex, hippocampus, and pons than the control diet group (Figure 1E). In addition, higher soluble Aβ42 levels in the hippocampal regions are shown in the Late-Def group (Figure 1F), and insoluble Aβ42 levels increase in the cortex, hippocampus, and pons of the Late-Def group, compared to the control group (Figure 1G).

### 3.2. Vitamin D Deficiency Accelerated the Pathological Aβ Process and Reduced Aβ Degradation

**Figure 2:**
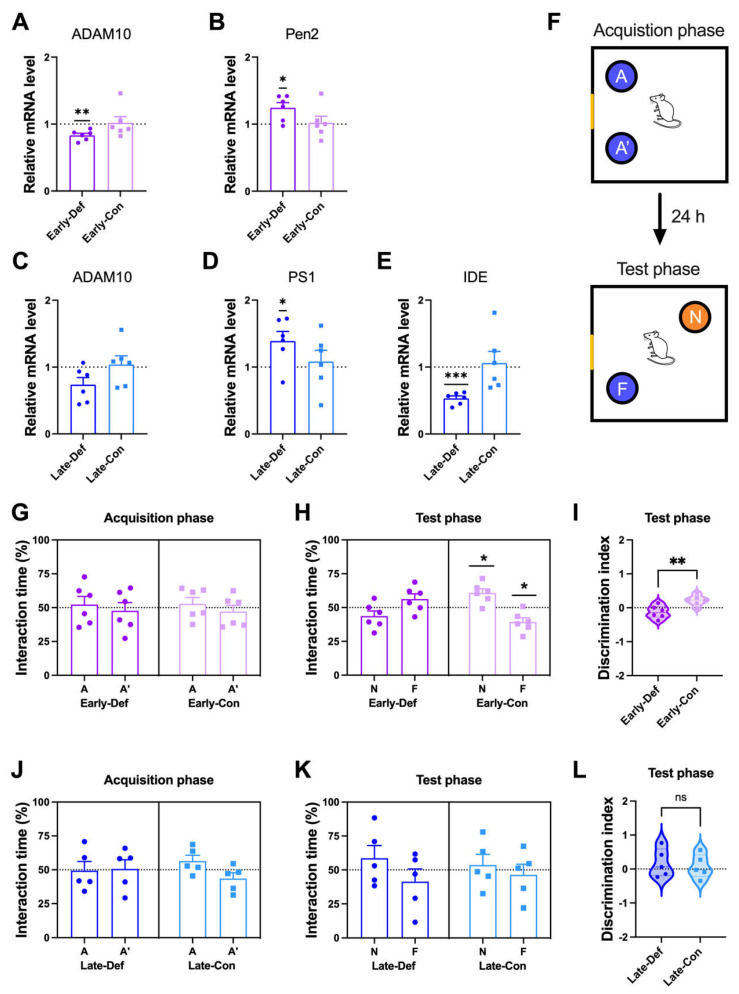
Vitamin D deficiency induced more Aβ processing and memory impairment in AD model mice. (**A**,**B**) Results of a quantitative real-time polymerase chain reaction (qRT-PCR) analysis of RNA for the key factors of Aβ processing of (**A**) ADAM10 and (**B**) Pen2 in the Early-Def (*n* = 6) and Early-Con (*n* = 6) groups are shown. (**C**–**E**) The qRT-PCR analysis results of (**C**) ADAM10, (**D**) PS1, and (**E**) IDE of the Late-Def (*n* = 6) and Late-Con (*n* = 6) groups are shown. (**F**) Schematic diagram of the novel place recognition (NPR) task. A and A’ denote identical objects. F and N indicate familiar and novel locations. A Yellow fragment in the rectangular outline represents a mark on the wall as a visuospatial cue. (**G**) Object interaction time of the Early-Def and Early-Con groups during the acquisition trial. (**H**) Interaction time in a novel location and familiar location of the Early-Def and Early-Con groups. (**I**) The discrimination index implies the relative preference for the novel location. The interaction times of the Late-Def and Late-Con groups in (**J**) the acquisition and (**K**) test phases are illustrated. (**L**) The discrimination index of mice in the Late-Def and Late-Con groups is shown. Dots represent individual mice data in each group. The interaction times for each location are expressed as a percentage of the total exploration time and as the statistical difference against a 50% theoretical mean (one-sample *t*-test). The data are presented by the mean ± SEM of each group. * *p* < 0.05, ** *p* < 0.01, and *** *p* < 0.001 by the Student’s *t*-test.

To investigate the reasons for the increase of the Aβ level in a vitamin D-deficient state, we examined the transcriptional level of the α-secretase gene in brain tissue, including the prelimbic area, thalamus, and cerebellum. ADAM10 has been identified as the constitutive α-secretase involving the non-amyloidogenic process. The vitamin D-deficient state diminished ADAM10 expression in the early and late stages (Figure 2A,C, respectively).

To evaluate whether vitamin D deficiency has a direct effect on Aβ generation, we measured the amyloidogenic process-related gene expression. The cleavage of APP by γ-secretase is the second step in the amyloidogenic process of Aβ generation. Vitamin D deficiency at the early stage induced more expression of PEN2 (Figure 2B), and deficiency at the late stage induced more PS1 gene expression (Figure 2D). PEN2 and PS1 consist of subunits of γ-secretase. In addition, the Late-Def group had lower mRNA levels of IDE, an Aβ-degrading enzyme, compared to the control group (Figure 2E).

### 3.3. Vitamin D Deficiency in the Early Stage Induced Memory Impairment

Working spatial memory was assessed using the NPR test (Figure 2F). In the acquisition phase, both the Early-Def and Early-Con groups showed no difference in interaction timed between two places: A and A′ (Figure 2G). In the test phase, only Early-Con showed more time spent in a novel location with normal memory function (Figure 2H). In addition, there were significant differences in the discrimination index between the two groups (Figure 2I). Vitamin D depletion at a young age is associated with reduced working memory. Vitamin D deficiency in the early stage also induced anxiety-like behaviors in the elevated plus-maze test (Figure S2A–F).

In the acquisition phase, both the Late-Def and Late-Con groups did not distinguish the two locations (Figure 2J). In addition, both groups displayed similar interaction times spent in both locations, with memory impairment in the test phase (Figure 2K). Moreover, there were no differences between the two groups in the discrimination index (Figure 2L). The Late-Con was given a behavioral test at ~6.5 months old, and these 5xFAD mice already exhibited 4- to 5-month-old memory impairment [33]. Thus, these results were derived from the floor effects of memory impairment in the AD model mice.

### 3.4. Vitamin D Injection in the Late-Stage Ameliorated the Brain Aβ Load

**Figure 3:**
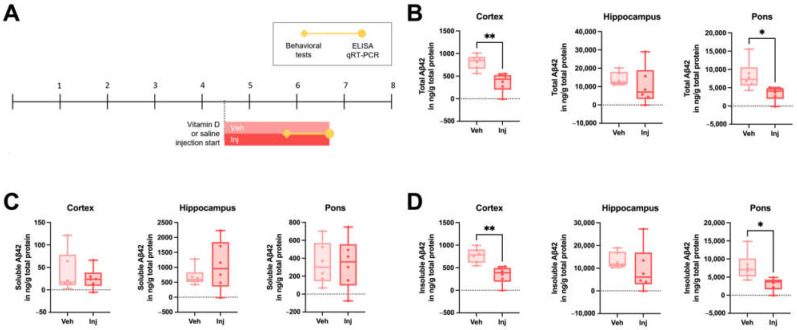
Vitamin D supplementation in the late stage decreased the Aβ load in AD model mice. (**A**) Experimental scheme of the vitamin D-supplemented state induced by the intraperitoneal injection of vitamin D in 5xFAD mice. At 4.5 months old, Veh (*n* = 6) and Inj (*n* = 6) group mice were administered vitamin D or saline, respectively. (**B**) The total Aβ42 levels in the cortex, hippocampus, and pons of the ~7-month-old Veh and Inj groups were measured using ELISA kits. (**C**) The soluble and (**D**) insoluble Aβ42 levels are also shown. Dots represent the data on individual mice in each group. The data are presented by the mean ± SEM of each group. * *p* < 0.05 and ** *p* < 0.01 by the Student’s *t*-test.

Due to the observed etiologic effects of vitamin D deficiency on AD pathophysiology, we further explored the potential therapeutic effect of vitamin D on AD by generating a vitamin D supplementation state by the intraperitoneal injection of a vitamin D solution in 5xFAD mice starting at 4.5 months old (Figure 3A). The total Aβ42 levels were significantly lower in the cortex and pons of the vitamin D-injected group compared to the vehicle-injected group (Figure 3B). There were no significant differences in the soluble Aβ42 levels between the two groups (Figure 3C), but the Inj group exhibited significantly lower insoluble Aβ42 levels in the cortex and pons (Figure 3D). In addition, the intermediate stage of the vitamin D-injected AD model mice exhibited decreased Aβ42 levels compared to the vehicle groups (Figure S3B–D).

### 3.5. Vitamin D Injection Reduced the Pathological Aβ Process

**Figure 4:**
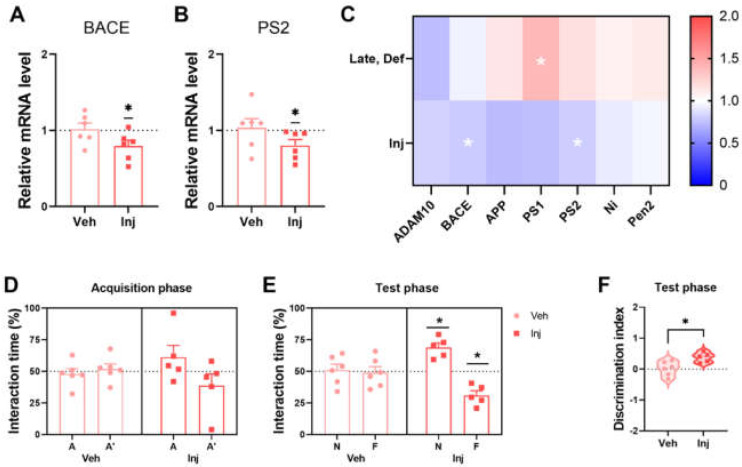
Vitamin D supplementation resulted in less Aβ processing and improved memory in AD model mice. (**A**,**B**) The results of the qRT-PCR analysis of mRNA expression of (**A**) BACE and (**B**) PS2 in the Veh (*n* = 6) and Inj (*n* = 6) groups are exhibited. (**C**) A heatmap of the qRT-PCR results of Aβ processing-related gene expression is illustrated to compare the Veh (*n* = 6) and Inj (*n* = 6) groups. The interaction time spent in each location of the Veh (*n* = 6) and Inj (*n* = 5) groups in (**D**) the acquisition phase and (**E**) test phase are illustrated. (**F**) The discrimination indexes of the Veh and Inj groups are shown. Dots represent the data of individual mice in each group. Interaction times are expressed for each location as a percentage of the total exploration time and were tested for the statistical difference against a 50% theoretical mean (one-sample *t*-test). The data are presented by the mean ± SEM of each group. * *p* < 0.05 by the Student’s *t*-test.

To assess the changes in Aβ generation, the mRNA levels of β-secretase and γ-secretase by qRT-PCR of the brain tissue were measured. Reduced levels of BACE mRNA expression in the vitamin D-injected group at the late stage are shown (Figure 4A). Moreover, the vitamin D injection encouraged less PS2 expression (Figure 4B), which is a subunit of γ-secretase. A heatmap analysis of the seven genes related to APP processing demonstrated that the six amyloidogenic process-related genes (BACE, APP, PS1, PS2, Ni, and PEN2) were upregulated in the vitamin D-deficient group and downregulated in the vitamin D-injected group compared to the Late-Con and Late-Veh groups, respectively (Figure 4C). Taken together, the data suggest that vitamin D attenuated the amyloidogenic process.

### 3.6. Vitamin D Injection in the Late-Stage Restored Normal Memory Function

Vitamin D injections improved the working memory in AD mice during the NPR test at the late stage. In the acquisition phase, the Veh and Inj groups showed ~50% interaction time in each location (Figure 4D). The Veh group could not distinguish the two locations following normal AD progression in the 5xFAD mice, whereas the Inj group showed discrimination in the test phase (Figure 4E). There was also a significant difference between the two groups in the discrimination index (Figure 4F). Therefore, the late-stage vitamin D injection is associated with restored working memory relative to the vehicle group. Moreover, the vitamin D supplementation attenuated anxiety-like behaviors in the open field test at ~5 months old (Figure S4A–C).

### 3.7. Vitamin D Deficiency Decreased Microglia-Related Aβ Clearance

**Figure 5:**
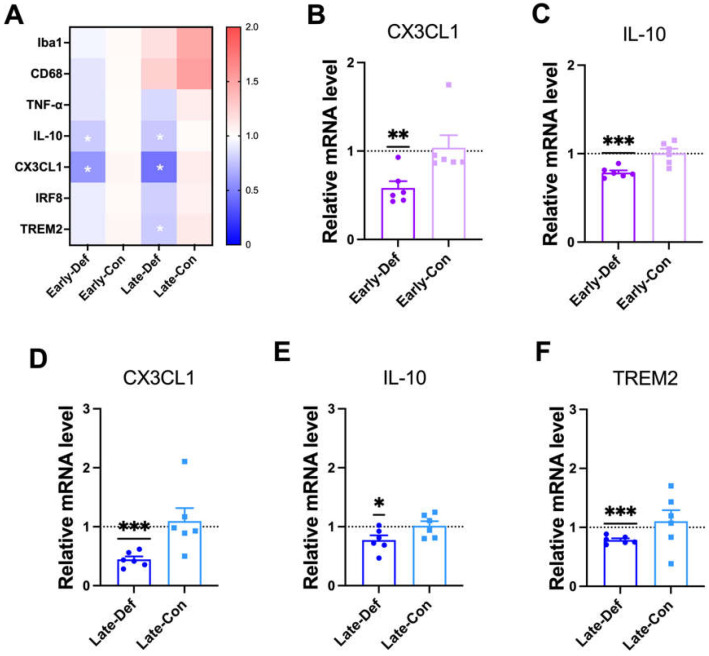
Vitamin D deficiency attenuated microglial activation and phagocytic responses. (**A**) A heatmap of qRT-PCR results for gene expression-related microglial function in Early-Def (*n* = 6), Early-Con (*n* = 6), Late-Def (*n* = 6), and Late-Con (*n* = 6) are shown. (**B**,**C**) The results from the qRT-PCR analysis of the mRNA expression of (**B**) CX3CL1 and (**C**) IL-10 in the Early- Def and Early-Con groups are shown. (**D**–**F**) The qRT-PCR results of the mRNA expression of (**D**) CX3CL1, (**E**) IL-10, and (**F**) TREM2 in the Late-Def and Late-Con groups are illustrated. Dots represent the data of individual mice in each group. The data are presented by the mean ± SEM of each group. * *p* < 0.05, ** *p* < 0.01, and *** *p* < 0.001 by the Student’s *t*-test.

Although vitamin D deficiency was shown to alter APP processing and reduced Aβ degradation, non-neuronal cells may also contribute to the amount of Aβ load. To explore this possibility, the transcription levels of the gene-related microglial functions in AD model mice were quantified. Briefly, in a heatmap of the qRT-PCR results, the Early-Def group showed decreased transcriptional levels of the IL-10 and C-X3-C motif Chemokine Ligand 1 (CX3CL1) genes compared to the Early-Con group. Furthermore, Late-Def also showed lower transcriptional levels of IL-10, CX3CL1, and triggering receptor expressed on myeloid cells 2 (TREM2) compared to the Late-Con group (Figure 5A). The majority of microglial function-related genes were downregulated in the Early-Def and Late-Def groups, but vitamin D supplementation did not alter the gene expression related to microglial function (Figure S5A–G).

CX3CL1, which is mediated in microglia migration and adhesion [39], was downregulated in the Early-Def group (Figure 5A) and Late-Def group (Figure 5D). IL-10, which is an inflammatory cytokine released by activated microglia, was also reduced by vitamin D deficiency in the early (Figure 5C) and late stages (Figure 5E). The Early-Def group revealed no significant difference in Ionized calcium-binding adapter molecule1 (Iba1), cluster of differentiation receptors (CD68), tumor necrosis factor α (TNF-α), interferon regulatory factor 8 (IRF8), and TREM2 expression compared to the Early-Con group (Figure S6A–E). Iba1 and CD68 are widely used markers of general microglial activation [40]. The late-Def group revealed a significantly lower mRNA level of TREM2 (Figure 5F), but there were no significant changes in the IRF8 gene mRNA level (Figure S6H). IRF8 and TREM2 genes are related to the capacity of microglial phagocytic responses [41,42].

### 3.8. Vitamin D Injection Attenuated Reactive Astrocytes

**Figure 6:**
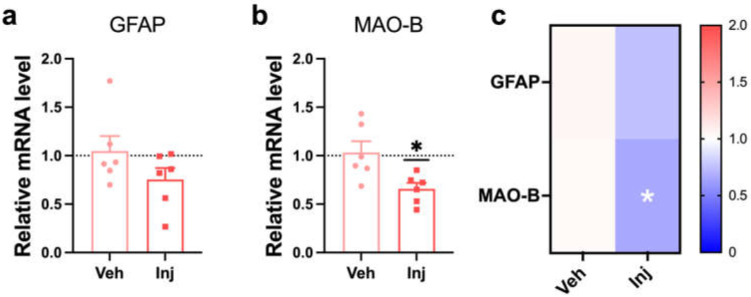
Vitamin D supplementation in a late-stage attenuated reactive astrocyte. (**A**,**B**) The qRT-PCR results from the analysis of the mRNA expression of (**A**) GFAP and (**B**) MAO-B in the Veh (*n* = 6) and Inj (*n* = 6) groups are shown. (**C**) A heatmap of the relative levels of mRNA expression related to a reactive astrocyte was used to compare the Veh (*n* = 6) and Inj (*n* = 6) groups. The data are presented by the mean ± SEM of each group. * *p* < 0.01 by the Student’s *t*-test.

We investigated the neurotoxic reactive astrocyte states in the vitamin D injection group to answer how vitamin D improves memory function in the late stage of AD in 5xFAD mice. Glial fibrillary acidic protein (GFAP) and monoamine oxidase B (MAO-B) were used as neurotoxic reactive astrocyte markers. The Inj group decreased GFAP mRNA expression but without statistical significance (Figure 6A). The MAO-B gene was significantly downregulated by the vitamin D injection (Figure 6B). In addition, the heatmap analysis also indicated a decrease in the markers for reactive astrocytes in the Inj groups compared to the Veh group. Moreover, vitamin D deficiency in the late stage did not alter the gene expression related to reactive astrocytes (Figure S7A,B).

## 4. Discussion

**Figure 7:**
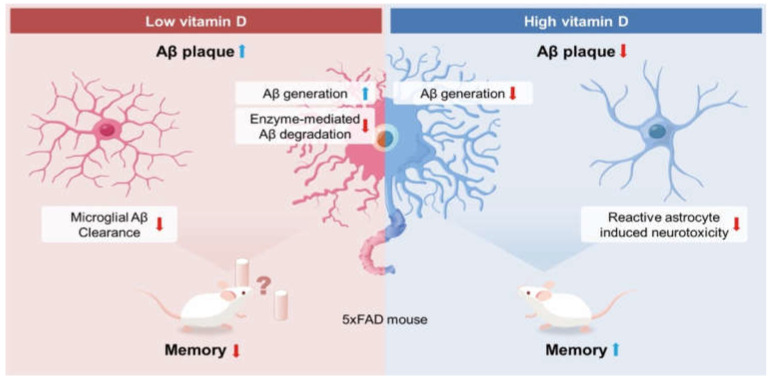
Possible mechanism for the observed effects of vitamin D on Alzheimer’s disease. Based on our results, low vitamin D induced increased Aβ generation and reduced enzyme-mediated Aβ degradation. Moreover, vitamin D deficiency attenuated the microglial clearance of Aβ, resulting in more Aβ load and memory impairment. On the other hand, according to the data, high vitamin D induced a lower Aβ load by reducing the Aβ generation and neurotoxic reactive astrocytes. In addition, vitamin D restored the visuospatial memory function in 5xFAD mice.

Our study found that vitamin D deficiency increased the Aβ load and memory impairment by increasing Aβ generation, decreasing enzyme-mediated degradation, and attenuating the microglial clearance of Aβ in 5xFAD mice (Figure 7). Along with the etiologic effects of vitamin D deficiency, we demonstrated that vitamin D supplementation in the late stage of AD resulted in a lower Aβ load and memory improvement with decreased Aβ generation and reactive astrocytosis. In analyzing our results, it is quite remarkable that vitamin D injections appear to have therapeutic effects on the memory function of the AD mouse model, even in the late stage.

Aside from its central role in calcium homeostasis and bone health, vitamin D has recently gained increased attention for its neuroprotective and anti-inflammatory functions and its critical role in brain development [43]. Moreover, epidemiological evidence demonstrates the relevance of vitamin D hypovitaminosis in the increased disease risk and progression of cognitive impairment and AD [44,45]. Moreover, recent reports suggest that low serum vitamin D levels are associated with decreased volumes in the hippocampal regions in elderly patients with cognitive decline [46]. Using the AD mouse model, we investigated the role of vitamin D deficiency in AD pathogenesis and cognitive function and found that a vitamin D-deficient state induced by diet led to a more significant brain Aβ load in both the early and late stages of AD progression. We observed the altered transcription levels of the genes related to Aβ formation, including ADAM10, BACE, PEN2, and PS2. This high Aβ load was caused by decreased α-secretase, increased γ-secretase, and decreased IDE levels. During Aβ anabolism, non-amyloidogenic APP processing is modulated by α-secretase, while amyloidogenic APP processing is modulated by β-secretase and γ-secretase. Based on these results, vitamin D deficiency appears to contribute significantly to elevated Aβ levels by attenuating the non-amyloidogenic process while also increasing the amyloidogenic process. In addition, IDE is an Aβ degradation enzyme, and its decrease in the deficient state also contributes to higher Aβ levels. Thus, the overall levels of Aβ could be changed by endogenous Aβ production and degradation enzymes. However, our results need to be interpreted carefully, as we only confirmed the transcriptional changes without investigating the protein levels and activities.

We examined the effects of vitamin D deficiency on spatial learning and memory function in the AD mouse model. The performance during the NPR task indicated spatial memory impairment in the vitamin D deficiency-induced early stage of AD progression. Vitamin D deficiency when the 5xFAD mice were young resulted in the faster onset of memory decline. Unfortunately, it is difficult to ascertain whether vitamin D deficiency caused memory impairment in the late stage, because the control group also showed memory impairment due to the floor effects of the NPR test. The Early-Def group displayed anxiety-like behaviors in the elevated plus-maze test. Anxiety symptoms are prevalent in AD patients [47], and anxiety is also a developmental precursor of AD [48]. In particular, anxiety was exhibited among patients with mild cognitive impairment, mild dementia, or symptoms of early-onset AD [49]. Therefore, vitamin D deficiency accelerates and worsens the pathogenic progression of AD.

Although vitamin D deficiency could be considered a risk factor in cognitive decline and AD, the effects of vitamin D supplementation on AD brains had not been thoroughly studied in animal models. Here, in exploring how vitamin D affects the mechanism of AD pathology, we noticed the potential therapeutic effects of vitamin D supplementation in mitigating AD progression in the 5xFAD AD mouse model, especially when considering that vitamin D intraperitoneal injections in the late stage reduced the total Aβ42 levels in the cortex and pons.

We also found that vitamin D reduced the Aβ load by decreasing β-secretase and γ-secretase in the amyloidogenic Aβ formation process. In cultured cells, others have reported that vitamin D and analogs induce α-secretase activity and increase the Aβ degradation enzyme NEP [24]. In this respect, vitamin D targets Aβ processing by reducing the amyloidogenic process while increasing both the non-amyloidogenic process and Aβ degradation. In addition to the ability of vitamin D to decrease the Aβ load, recent reports indicate that vitamin D also protects against Aβ-induced cytotoxicity and oxidative stress [47,50].

Memory impairment in the 5xFAD mouse model has been extensively characterized, and our 5xFAD mice showed memory impairment at 4 to 5 months old [33]. To investigate the therapeutic effects of vitamin D on AD, we started the vitamin D injection of 5xFAD mice from 4.5 months old and showed that vitamin D injection after the onset of AD symptoms could improve memory function, while the vehicle-injected groups showed memory impairment following the normal course of AD. Surprisingly, our results indicate that vitamin D is efficient in restoring spatial memory and reducing the Aβ load even when delivered after the onset of the major symptoms. In contrast, vitamin D injection in the intermediate stage did not show memory improvement but reduced anxiety-like behaviors in an open field test. This lack of significant memory improvement in the intermediate stage may be due to the possible result of the ceiling effect of the NPR test. Anxiety is one of the symptoms of AD, and a significant association between anxiety and AD risk was indicated by a meta-analysis [51]. Taken together, vitamin D supplementation may be beneficial in reducing anxiety and improving memory, depending on the disease stage of AD progression.

Besides Aβ pathology, AD is highly associated with prominent neuroinflammation [52], and the transgenic 5xFAD mouse model is characterized by predominant inflammatory and immune responses [53]. The microglia of the central nervous system (CNS) are resident macrophages that maintain and protect the CNS through phagocytosis and the clearance of pathogens [54]. In neurodegenerative diseases, including AD, microglia play disease-modifying roles by clearing away dying cells, neurons, dendrites, blood vessels, and amyloid plaques [55].

Consistent with a previous report [56], we found that vitamin D deficiency reduced IL-10, which is an anti-inflammatory cytokine, and this was further supported by the association with increased Aβ. Although vitamin D deficiency results in cytokine change, the causal relationship with increased Aβ is difficult to determine. If the direct effect of vitamin D deficiency on cytokine regulation precedes the Aβ increment, the suppressed anti-inflammatory cytokine, such as IL-10, can be interpreted as enhancing Aβ accumulation. On the other hand, the cytokine change may alter the Aβ-processing genes and result in an increase of Aβ peptides during vitamin D deficiency. Consequently, the increased Aβ, in turn, may trigger the neuroinflammatory process. Nevertheless, vitamin D deficiency worsens AD progression in 5xFAD mice.

We found that vitamin D-deficient mice showed reduced CX3CL1, IRF8, and TREM2 mRNA levels. It has been suggested that vitamin D deficiency increases Iba 1-labeled microglia [57], and vitamin D receptor activation inhibits microglia-mediated neuroinflammation and oxidative stress [28]. CX3CL1 (neurons)-CX3CR1 (microglia) signaling serves as a communication channel between neurons and microglia and plays a fundamental role in the regulation and maturation of cells [58]. A decline in the CX3CL1 levels due to vitamin D deficiency may be related to decreased Aβ clearance. IRF8-deficient microglia exhibit morphological alterations, as well as a reduced motility and phagocytic response [59]. The TREM2 gene is also related to the capacity of the microglial phagocytic responses, and the loss of TREM2 function leads to increased amyloid seeding [60]. In the vitamin D-deficient group, decreased CX3CL1, IRF8, and TREM2 mRNA levels correspond with reduced microglial phagocytic functions. In this context, an increased Aβ load in a vitamin D-deficient state can be interpreted as a consequence of decreased microglial phagocytic responses to Aβ with increased Aβ anabolism and reduced Aβ degradation pathways.

The prolonged activation of astrocytes and the release of proinflammatory cytokines, chemokines, and reactive oxygen and nitrogen species create a neurotoxic environment, which exacerbates AD progression [61,62]. On the other hand, vitamin D can suppress the activation of astrocytes with decreased GFAP-positive cells and increased BDNF-positive cells [63]. Likewise, we also found that vitamin D injections downregulate the MAO-B mRNA levels, which are increased in neurotoxically reactive astrocytes [64]. Vitamin D, an immunomodulator, ameliorates inflammation in the AD mouse model [65,66]. A recent report also found that reactive astrocytes generate an abundance of the inhibitory gliotransmitter GABA by MAO-B, resulting in memory impairment in the AD mouse model [67]. Decreased MAO-B levels in the vitamin D supplementation group imply attenuated neurotoxic reactive astrocytosis in the AD mouse model and relate to the rebound memory function after the onset of the main symptoms.

Here, we demonstrated that vitamin D dampened the neurotoxic reactive astrocytes, reduced the Aβ pathology, and improved the functional outcomes in the 5xFAD mouse model. Consequently, our data strongly suggest that vitamin D deficiency accelerates AD disease development, whereas vitamin D supplementation induces a reduced pathological process and functional impairment even in the late phase. Based on these results, we believe that vitamin D supplementation can be translated into clinical application for reducing amyloidopathy and gliopathy by controlling the transcription levels of the related enzymes.

Limitations:The findings of this study need to be interpreted carefully, considering some limitations. First, the normal diet based on the NIH-41 Open Formula was used as the control group in the vitamin D deficiency study. The standard diet and the vitamin D deficiency diet (TD.89123, ENVIGO, Indianapolis, USA) differed in their fat, carbohydrate, and protein compositions. This difference potentially affected the results as a confounding factor. Second, we only measured the transcription levels of the genes related to Alzheimer’s disease pathology to examine the pathologic and therapeutic roles of vitamin D since vitamin D regulates the transcriptional levels via binding to intracellular receptors and acting as transcription factors inside the nucleus. With the quantification of the related protein levels and activities, the implication of vitamin D deficiency and supplementation should have been more compelling. In future research, it is necessary to evaluate the protein levels and activities. Third, we conducted the novel place recognition test for assessing visuospatial memory. It could be more desirable to examine other domains of cognitive memory, such as working memory. Fourth, there is a concern about using different brain regions for Aβ and mRNA measurements. Indeed, we measured the Aβ levels in the cortex, hippocampus, and pons by ELISA and performed qRT-PCR with the homogenates of the rest of the brain areas to identify transcriptional changes in the genes related to Alzheimer’s disease pathology. This might be a crucial limitation in the interpretation of our data. Nevertheless, according to Oakley et al. [33], as the mice aged, the plaques were observed in a wide range of the brain, including the thalamus, brainstem, and olfactory bulbs. Our speculation that the overall levels of Aβ accumulation were proportional to those of the Aβ-measured areas should be confirmed in a further study. Fifth, the potential confounding factors that vitamin D deficiency may induce, such as bone changes or pain, were not completely controlled. Although we did not measure bone changes or pain directly, we analyzed the locomotor function as a surrogate variable for musculoskeletal pain. The total travel distance in the open field test did not differ between the vitamin D-deficient and normal diet groups (data not shown), implying that the locomotor function was not changed by vitamin D deficiency. Additionally, measuring the vitamin D levels in the experimental mice must be more informative. Nevertheless, based on previous reports [68,69,70,71,72], we believed that administrating the vitamin D-deficient diet for more than six weeks could reliably cause vitamin D deficiency. In the future, examining the objective signs of hypovitaminosis D will be desirable.

## 5. Conclusions

The molecular mechanism underlying the pathogenic and therapeutic effects of vitamin D on AD are not yet fully understood. Therefore, to better understand how vitamin D regulates the expression of the genes related to AD pathology, we induced vitamin D deficiency in 5xFAD mice by providing a vitamin-D-deficient diet and observed the changes in the mRNA levels of the genes related to Aβ processing, which resulted in an increase in the Aβ load in the brain with corresponding memory impairment in the AD mouse model. In addition, vitamin D supplementation enhanced the memory function by ameliorating amyloidopathy and gliopathy in the AD mouse model. By understanding this mechanism, especially in regards to the nutritional factors affecting AD, we propose a further investigation of vitamin D’s role as a potential therapeutic treatment for AD patients.

## Figures and Tables

**Table 1:** List of primers used for qRT-PCR.

Gene Name	Orientation	Sequence
GAPDH	Forward	GGAGAAACCTGCCAAGTATG
	Reverse	CATACCAGGAAATGAGCTTGAC
BACE	Forward	AACGAATTGGCTTTGCTGTC
	Reverse	AGCCACAGTCTTCCATGTCC
ADAM10	Forward	CTTCGCCGTTTCTCCTG
	Reverse	CCAGGAGAGGAGCAGAA
APP	Forward	GAACTACATCACCGCTGTGC
	Reverse	CGCGGACATACTTCTTTAGC
PS1	Forward	GGTCGTGGCTACCATTAAGTC
	Reverse	GCCCACAGTCTCGGTATCTT
PS2	Forward	CGCTGCTACAAGTTCATCCA
	Reverse	TGAGCACTTCCCCAAGGTAG
Ni	Forward	CACTATGTGCCATGCAGCTC
	Reverse	GCTTGATGCTGAAGGTGCTT
Pen2	Forward	ATTGAACCTGTGCCGGAAGT
	Reverse	GCCTCTCGGAAGAACCACAA
Iba1	Forward	GTCCTTGAAGCGAATGCTGG
	Reverse	CATTCTCAAGATGGCAGATC
CD68	Forward	TTCACCTTGACCTGCTCTCTC
	Reverse	GTAGGTTGATTGTCGTCTGCG
TNF-α	Forward	TCTTCTGTCTACTGAACTTCGG
	Reverse	AAGATGATCTGAGTGTGAGGG
IL-10	Forward	CGGGAAGACAATAACTGCACCC
	Reverse	CGGTTAGCAGTATGTTGTCCAGC
CX3CL1	Forward	CAGCATCGACCGGTACCTT
	Reverse	GCTGCACTGTCCGGTTGTT
GFAP	Forward	TCCTGGAACAGCAAAACAAG
	Reverse	CAGCCTCAGGTTGGTTTCAT
MAO-B	Forward	TACTTGGGGACCGAGTGAAGCT
	Reverse	CCAAAGCAGGTGGAATGGCACT
IRF8	Forward	GGATATGCCGCCTATGACACA
	Reverse	CATCCGGCCCATACAACTTAG
NEP	Forward	GATCAGCCTCTCGGTCCTTG
	Reverse	TGTTTTGGATCAGTCGAGCAG
IDE	Forward	CAAACCTCTCCTTCCAAGTCAGC
	Reverse	TGTTCTCCGAGGTGCTCTGCAT
TREM2	Forward	CTACCAGTGTCAGAGTCTCCGA
	Reverse	CCTCGAAACTCGATGACTCCTC

## Data Availability

All data generated or analyzed during this study are included in this published article and its supplementary information files. The data presented in this study are available upon reasonable request from the corresponding author.

## Supplementary Materials

The following supporting information can be downloaded at https://www.mdpi.com/article/10.3390/biomedicines10081824/s1: Figure S1: Weight gain was higher in the Early-Def group. Figure S2: Early-Def showed increased anxiety-like behaviors. Figure S3: Intermediate VitD showed a decreased Aβ load. Figure S4: Intermediate VitD showed decreased anxiety-like behaviors. Figure S5: Vitamin D supplementation in the late phase did not alter the gene expression-related microglial function. Figure S6: The qRT-PCR results of the vitamin D deficiency experiment are shown. Figure S7: Vitamin D deficiency in the late phase did not alter the gene expression-related reactive astrocyte.
