# Supplementary material for: The Role of Vitamin D in Alzheimer’s Disease: A Transcriptional Regulator of Amyloidopathy and Gliopathy
Source: Biomedicines. 2022 Jul 28;10(8):1824. doi: 10.3390/biomedicines10081824 (PMC9404847; doi:10.3390/biomedicines10081824)
Supplement: Supplementary file 1 [file biomedicines-10-01824-s001.zip › biomedicines-1771016-supplementary.pdf]

Supplementary Materials

Table of Contents

- Supplementary Methods
- Figure S1. Early-Def showed increased anxiety-like behaviors
- Figure S2. Intermediate, VitD showed decreased Aβ load
- Figure S3. Intermediate, VitD showed decreased anxiety-like behaviors
- Figure S4. Vitamin D supplementation in the late phase did not alter the gene expression-related microglial function
- Figure S5. Vitamin D deficiency in the late phase did not alter the gene expression-related reactive astrocyte

Supplementary Methods

Mice

To assess the effects of vitamin D supplementation, we divided the mice into two groups (n = 6/group) in supplementary data.: i) The intermediate-vitamin D supplementation group mice were intraperitoneally injected each week with 410 ng/g cholecalciferol (PHR1237, Sigma-Aldrich, Burlington, MA, USA) in saline containing 1% ethanol from 3 months old. ii) The Intermediate, Vehicle group mice were injected intraperitoneally only saline containing 1 % ethanol (injection volume: individual body weight (g) / 100 mL saline) from 3 months old. All the mice were supplied with food and water *ad libitum* under 12 h light/dark cycles (lights on at 19:00 pm) with constant temperature and humidity of 20 ± 2 ℃ and 55 ± 5 %.

Behavioral test

Animals were tested during the dark period (between 19:00 and 24:00 pm), which is the active period for nocturnal animals. An open field test (OFT) was used to evaluate the locomotor activity and anxiety levels of mice exposed to a novel environment. The test chamber (40 × 40 × 40 cm) was located in a sound-attenuated testing room under red light (~50 lux). Mice were gently placed in the center of the empty test chamber and allowed to move freely for 10 min. The tests were conducted twice with an inter-trial interval of 24 h. While the first trial was used to assess anxiety about novel environments and open spaces, the second one was analyzed to assess locomotor functions. The total distance, entries in each zone, and times spent in each zone were analyzed using an automated video tracking system Smart3 (Panlab, Harvard Apparatus, Barcelona, Spain).

An elevated plus maze (EPM) test was conducted using an apparatus that consisted of four arms (25 cm long × 5 cm wide) connected to a center zone (5 × 5 cm). The maze was elevated 50 cm

above the floor. Two of the arms were enclosed with walls (16 cm high, closed arms), and the other arms had no border in place of the walls (open arms). The apparatus was illuminated ~300 lux. A mouse was placed on one of the open arms with its head facing toward the center zone. The test was video-recorded for 5 min. The entries into the open and closed arm, time spent in each arm, and distance traveled in each arm were assessed for anxiety-like behavior.

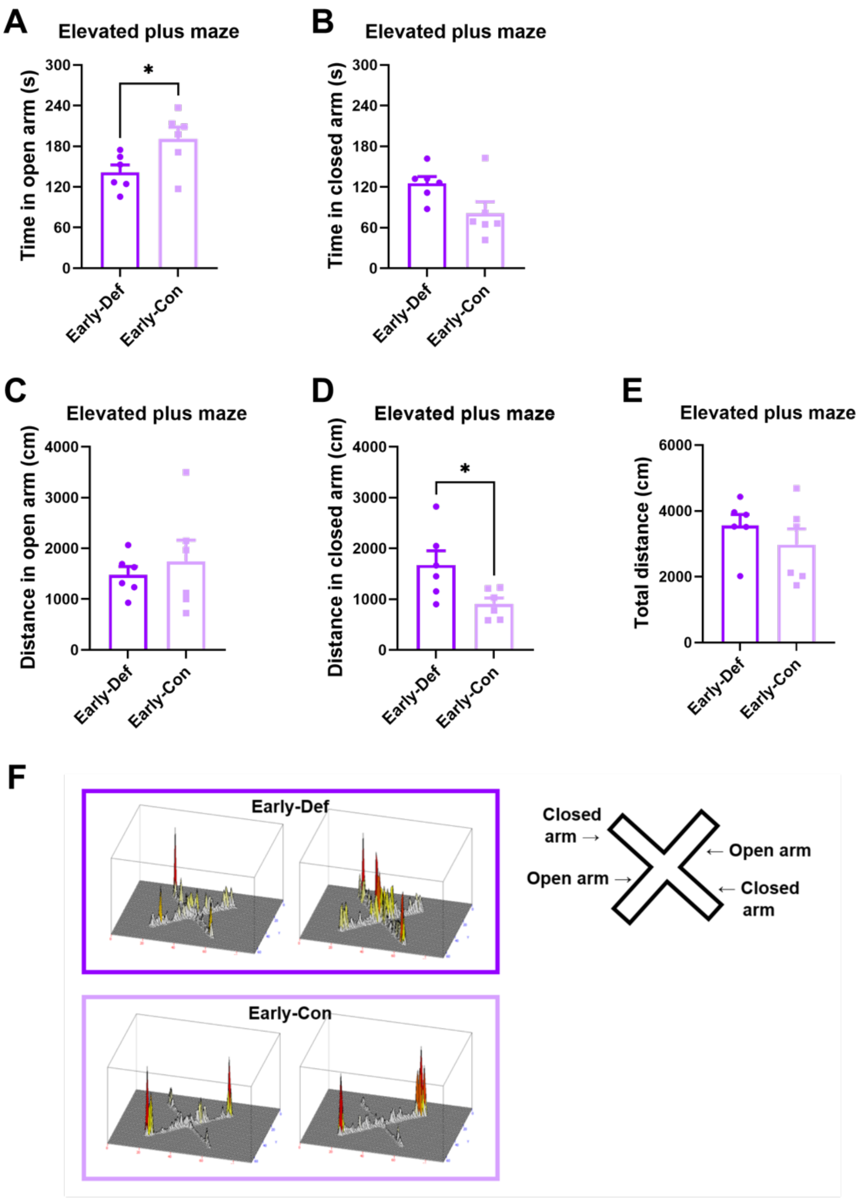

Figure S1. Early-Def showed increased anxiety-like behaviors. (**A**) Time spent in open arm of elevated plus maze (EPM) test of Early-Def (n=6) and Early-Con (n=6) groups are shown. (**B**) Time spent in closed arm are illustrated. (**C, D**) Distance in open arm and closed arm are presented. (**E**) Total distance are indicated. (**F**) Representative 3D heatmaps of activity in EPM apparatus are presented. Dots represent data on individual mice in each group. The data are presented by mean ± SEM of each group. *$p < 0.05$ by Student's t-test.

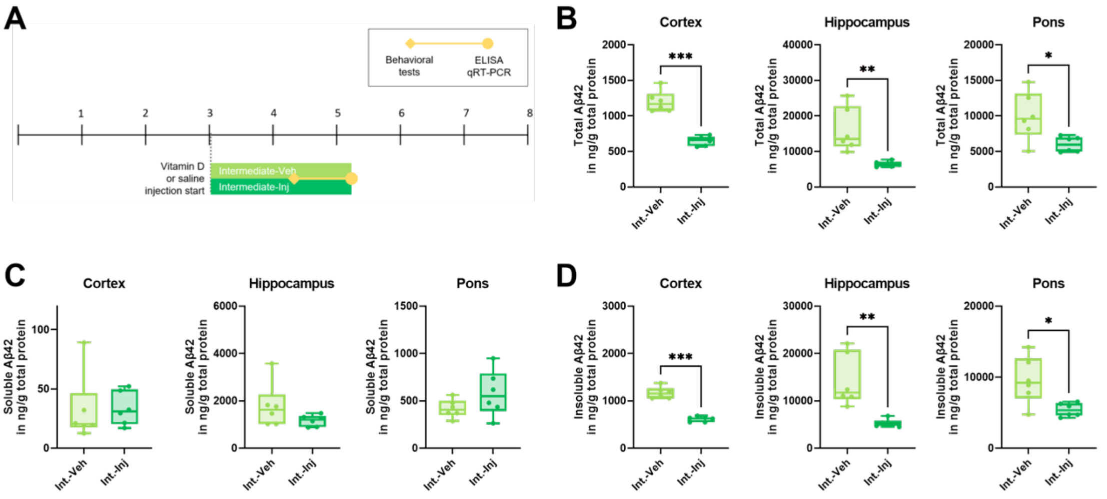

Figure S2. Intermediate, VitD showed decreased Aβ load. (**A**) An experimental scheme of vitamin D supplemented state by intraperitoneal injection of vitamin D in 5xFAD mice was illustrated. 3 months old Int.-Veh (n=6) and Int.-Inj (n=6) group mice were started to administrated vitamin D or saline, respectively. (**B**) The total Aβ42 levels in the cortex, hippocampus, and pons of ~5 months old Int., Veh and Int., Inj groups were measured using ELISA kits. (**C**) Soluble and (**D**) insoluble Aβ42 levels were also represented. Each dot represents individual mice data in each group. Each dot represents individual mice data in each group. Interaction time of each location is expressed as a percentage of total exploration, and statistical difference against 50 % theoretical mean (one-sample t-test) was indicated. Dots represent data on individual mice in each group. The data are presented by mean ± SEM of each group. *$p < 0.05$, **$p < 0.01$, and ***$p < 0.001$ by Student's t-test.

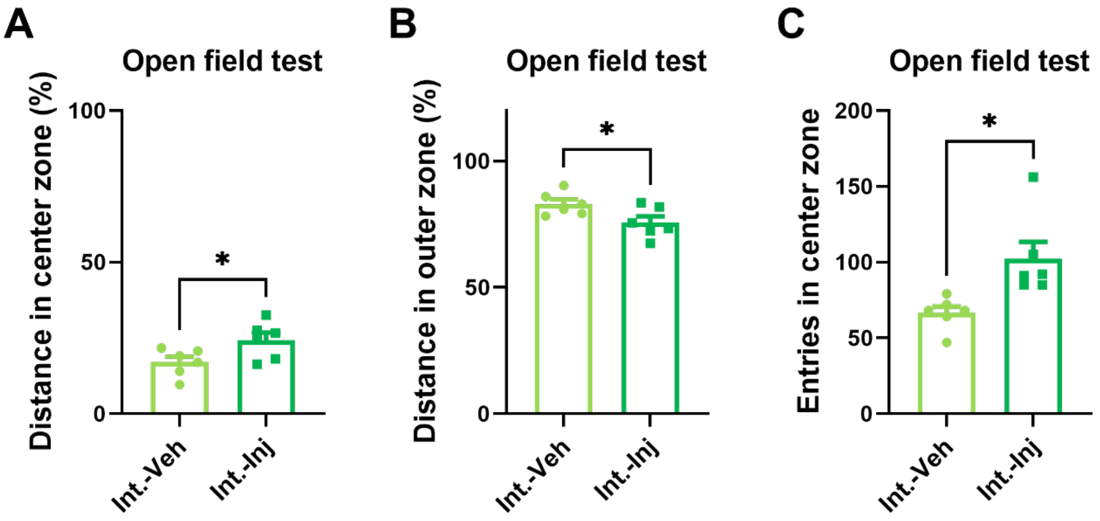

Figure S3. Intermediate, VitD showed decreased anxiety-like behaviors. (**A, B**) Distance in the center zone and outer zone of the open field test of ~ 5 months old Int.-Veh (n=6) and Int.-Inj (n=6) group are represented. (**C**) Entries in the center zone are shown. Dots represent the data of individual mice in each group. The data are presented by mean ± SEM of each group. *$p < 0.05$ by Student's t-test.

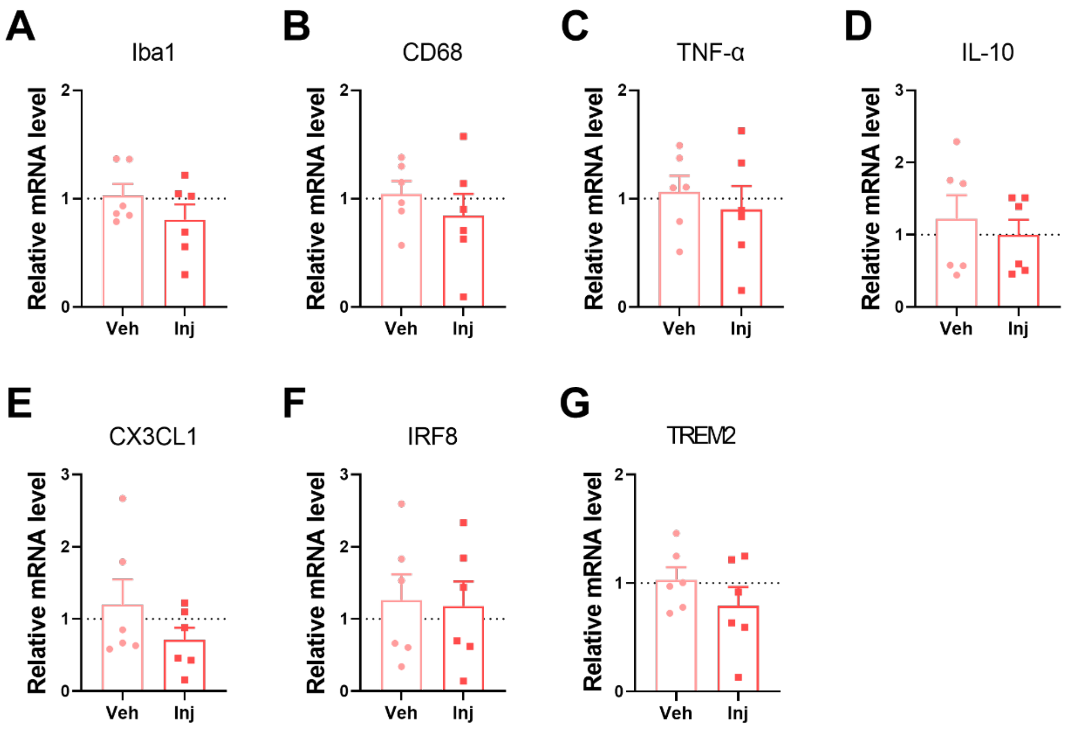

Figure S4. Vitamin D supplementation in the late phase did not alter the gene expression-related microglial function. qRT-PCR analysis of mRNA expression of (**A**) Iba1, (**B**) CD68, (**C**) TNF-α, (**D**) IL-10, (**E**) CX3CL1, (**F**) IRF8 and (**G**) TREM2 in Veh (n=6) and Inj (n=6) groups are shown. Dots represent the data of individual mice in each group. The data are presented by mean ± SEM of each group. *p < 0.05 by Student's t-test.

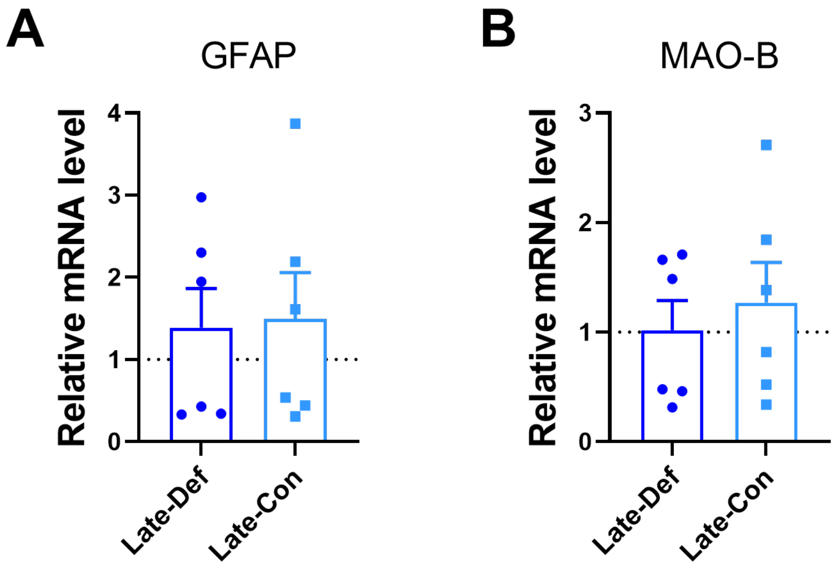

Figure S5. Vitamin D deficiency in the late phase did not alter the gene expression-related reactive astrocyte. (**A, B**) qRT-PCR analysis of mRNA expression of (**A**) GFAP and (**B**) MAO-B in Late-Def (n=6) and Late-Con (n=6) groups are shown. Dots represent the data of individual mice in each group. The data are presented by mean ± SEM of each group. *$p < 0.05$ by Student's t-test.
